# A systematic review of the biological mechanisms linking physical activity and breast cancer

**DOI:** 10.20463/pan.2020.0018

**Published:** 2020-09-30

**Authors:** Bok Sil Hong, Kang Pa Lee

**Affiliations:** 1 Life Science Research Center, Cheju Halla University, Jeju, Republic of Korea; 2 Department of Nursing, Cheju Halla University, Jeju, Republic of Korea; 3 Research & Development Center, UMUST R&D Corporation, Seoul, Republic of Korea

**Keywords:** exercise, breast cancer, metabolic hormones, inflammatory markers, myokines, adipokines, stress hormones, ROS

## Abstract

**[Purpose]:**

Epidemiological evidence has shown that leisure-time physical activity and structured exercise before and after breast cancer diagnosis contribute to reducing the risk of breast cancer recurrence and mortality. Thus, in this review, we aimed to summarize the physical activity-dependent regulation of systemic factors to understand the biological and molecular mechanisms involved in the initiation, progression, and survival of breast cancer.

**[Methods]:**

We systematically reviewed the studies on 1) the relationship between physical activity and the risk of breast cancer, and 2) various systemic factors induced by physical activity and exercise that are potentially linked to breast cancer outcomes. To perform this literature review, PubMed database was searched using the terms “Physical activity OR exercise” and “breast cancer”, until August 5th, 2020; then, we reviewed those articles related to biological mechanisms after examining the resulting search list.

**[Results]:**

There is strong evidence that physical activity reduces the risk of breast cancer, and the protective effect of physical activity on breast cancer has been achieved by long-term regulation of various circulatory factors, such as sex hormones, metabolic hormones, inflammatory factors, adipokines, and myokines. In addition, physical activity substantially alters wholebody homeostasis by affecting numerous other factors, including plasma metabolites, reactive oxygen species, and microRNAs as well as exosomes and gut microbiota profile, and thereby every cell and organ in the whole body might be ultimately affected by the biological perturbation induced by physical activity and exercise.

**[Conclusion]:**

The understanding of integrative mechanisms will enhance how physical activity can ultimately influence the risk and prognosis of various cancers, including breast cancer. Furthermore, physical activity could be considered an efficacious non-pharmacological therapy, and the promotion of physical activity is probably an effective strategy in primary cancer prevention.

## INTRODUCTION

Breast cancer is one of the most common malignant tumors worldwide, accounting for 30% of all new cases of female cancers [1]. The incidence rate of breast cancer has risen slightly by approximately 0.3% per year, whereas the 5-year survival rate of female breast cancer patients is 90%, which is higher than that of all cancer patients (67%, in average). Approximately 5%–10% of breast cancer cases can be attributed to an inherited genetic predisposition with a family history, such as mutations in two high-penetrance tumor suppressor genes, breast cancer gene 1 and 2 [2]. However, breast cancer is more frequently associated with environmental, reproductive, and lifestyle factors, including nutrition and physical activity [3], that may play an essential role in the pathogenesis of breast cancer.

In recent years, epidemiological studies have been conducted on the relationship between physical activity and cancer outcomes, demonstrating a protective role of physical activity in breast cancer [4-8]. Leisure-time physical activity is associated with lower risks of 13 types of cancer [5], and exercise-associated reduction in breast cancer risk has been apparent in early-stage breast cancer patients [6]. In addition, high-risk breast cancer patients meeting the minimum Physical Activity Guidelines for Americans [9] experienced 50% reduced hazards of recurrence and mortality [7]. Therefore, epidemiological evidence supports that leisure-time physical activity and structured exercise before and after breast cancer diagnosis contribute to reducing the risk of breast cancer recurrence and mortality.

**Figure 1.**
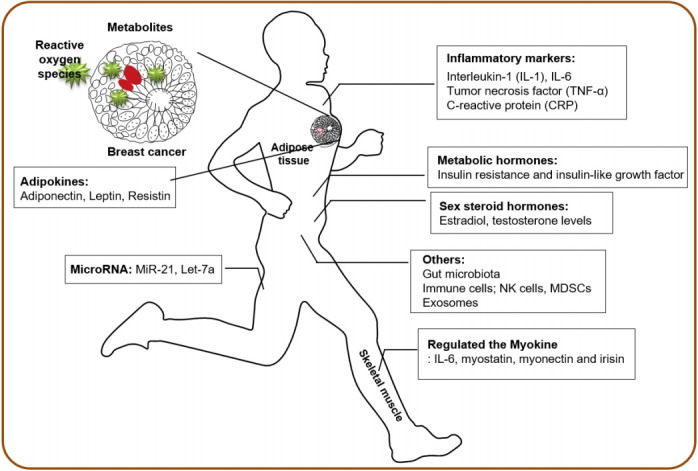
Physical activity-induced systemic factors associated with breast cancer outcomes

Exercise can substantially influence whole-body homeostasis by affecting multiple organ systems, and the integrative biology of exercise in skeletal muscle adaptation has been extensively studied using omics approaches aiming to decipher the molecular basis of exercise responses [10-12]. In addition, multiple biological mechanisms at systemic levels are hypothesized to mediate the potential protective effects of physical activity and exercise on cancer prevention [13-16]. The current review summarizes the physical activity-dependent regulation of systemic factors that may influence breast cancer progression and clinical outcomes (Figure 1; Table 1).

## The biological mechanisms linking physical activity and breast cancer

### Sex steroid hormones

Women with elevated systemic levels of estrogens and androgens have increased risks of breast cancer incidence and development [17,18]. In premenopausal women, physical activity is inversely correlated with sex hormones, estradiol, and testosterone level [19,20]. As sex hormones in postmenopausal women are primarily produced in the adipose tissue, physical activity has been associated with decreased estrone and estradiol levels after adjustment for Body Mass Index [21,22], indicating that weight loss and lower adiposity are linked to controlled sex hormone levels and lower risk of breast cancer. A meta-analysis of the effect of physical activity on sex hormones demonstrated that physical activity produced protective effects against breast cancer by decreasing the levels of circulating sex hormones, regardless of menopausal status and weight loss [23].

### Metabolic hormones

Several studies have shown that elevated plasma insulin levels are associated with an increased incidence of various cancers [24] and higher recurrence in breast cancer survivors [25,26]. Insulin resistance and insulin-like growth factor (IGF) may increase the risk of breast cancer by increasing the level of circulating estrogen [27]. Exercise can reduce insulin levels and insulin resistance, thereby decreasing fasting glucose, total IGF-1, and increasing IGF binding proteins [28]. In addition, after exercise interventions, breast cancer patients have reduced fasting insulin levels due to the reductions in body weight, anticipating better prognosis of breast cancer [29]. However, most of the benefits of exercise-induced modulation might be associated with body weight loss and comorbidities, such as type 2 diabetes and metabolic syndrome [30,31].

### Inflammatory markers, cytokines, and adipokines

Cancer-related inflammation is one of the hallmarks of cancer [32], and interleukin-1 (IL-1), IL-6, tumor necrosis factor alpha (TNF-α), and C-reactive protein (CRP) are widely recognized as biomarkers of systemic inflammation related to breast cancer [33]. Studies have shown that increased levels of pro-inflammatory cytokines and CRP have been linked to increased cancer risk and reduced overall survival of breast cancer [34,35]. Physical activity generally has an anti-inflammatory effect and reduces systemic inflammation in healthy individuals without cancer diagnosis. The impact of physical activity on the levels of IL-6, TNF-α and CRP varies and has its limitations. Further, a meta-analysis of exercise intervention showed no effect on the levels of CRP, IL-6, or TNF-α [31].

Adipose tissues, bearing one of the multiple cell types in the mammary gland, secrete adiponectin, leptin, resistin, and other cytokines. The modulation of circulating adipokines by physical activity has also been demonstrated [36]. Leptin stimulates growth, migration, and invasion of breast cancer via its pro-inflammatory effect, whereas adiponectin is an anti-inflammatory factor and is inversely associated with adiposity. Decreased levels of adiponectin are associated with higher body mass indices and higher fat percentages, whereas the ratio of adiponectin to leptin is a key determinant of the effect of adipokines on the pathological process of breast cancer [37,38]. Significant elevations in serum adiponectin levels and reductions in serum leptin levels have been observed with physical activity interventions by directly lowering the amount of body fat [39,40]. Therefore, physical activity may regulate inflammatory cytokines and adipokines; however, the reduced risk of breast cancer is strongly associated with fat mass and weight loss.

### Myokines and stress hormones as exercise factors

Skeletal muscle, the largest organ in our body, secretes numerous myokines, such as IL-6, myostatin, myonectin, and irisin, and the circulating myokines levels are regulated by physical exercise [41]. A large-scale omics-based approach is aimed at elucidating the entire secretome secreted by muscle cells to understand the molecular basis of exercise adaptation. Preclinical studies have demonstrated that irisin, which increases with physical activity, can inhibit breast cancer viability due to increased caspase activity and suppressed NF-κB activity [42].

The stress hormones catecholamines are exercise factors responsible for breast cancer progression inhibition. Plasma epinephrine and norepinephrine rapidly increase by exercise [43]; in contrast, cortisol levels are dependent on the duration and intensity of exercise [44]. Exercise-induced catecholamines mediate breast cancer suppressive effects by activating the tumor suppressor Hippo signaling pathway [45]. However, data on the role of stress hormones as exercise factors have not been studied directly in breast cancer patients, and they could induce an opposite effect on breast cancer protection [46].

### Other systemic factors

In addition to hormones, inflammatory markers, and myokines, several other circulating systemic factors are regulated by physical activity and exercise. Various plasma metabolites are related to physical activity, and these metabolites may play an essential role in the protective effect of cancer progression. Several studies have shown that plasma metabolites are associated with the risk of breast cancer and have potential as biomarkers for the early diagnosis of breast cancer [47,48].

Oxidative stress and reactive oxygen species have been implicated in a number of diseases as well as the initiation and progression of cancer [49]. Interestingly, acute exercise produces pro-oxidant environments; however, repeated exercise stimulates antioxidant defenses, resulting in a greater capacity to resist oxidative environments [50]. In this context, the effect of repeated physical activity on oxidative stress may be beneficial for preventing the progression and metastasis of breast cancer.

Recent studies have shown that the expression levels of circulating microRNAs are modulated by physical activity and exercise in healthy individuals and patients with various diseases [51-53]. Because of their pivotal role in controlling cell proliferation, microRNAs may be important regulators of exercise adaptation and potential biomarkers of exercise response [51,52]. The expression of several breast cancer-related microRNAs, such as miR-21 and let-7a, is altered by exercise [53], and thereby circulating microRNAs may be mediators of the association between physical activity and breast cancer.

In addition to various soluble mediators, extracellular vesicles containing functional molecules such as proteins, lipids, mRNA, and microRNAs can exert systemic biological effects by providing the means for inter-tissue crosstalk during physical exercise [54]. Physical exercise triggers a rapid release of extracellular vesicles into the circulation [55], and their compositions via delivery of myokines play an essential role in exercise adaptation throughout the body [56]. Therefore, extracellular vesicles could mediate the beneficial effects of exercise [57] and potentially affect breast cancer progression.

Physical activity has an impact on the level and activity of circulating immune cells, such as natural killer cells, which are the most responsive immune cells to exercise-dependent mobilization to the circulation [58]. The mobilization of IL-6-sensitive natural killer cells was significantly increased by epinephrine in mice that experienced voluntary wheel running, resulting in the reduction of tumor incidence and growth [59]. In addition, myeloid-derived suppressor cells, as regulators of the immune system in the tumor microenvironment, could regulate tumor growth and metastasis by controlling inflammatory markers with exercise and weight loss [60].

Finally, emerging evidence suggests that the gut microbiome may confer susceptibility to several cancers and may influence the therapeutic responses, suggesting that the microbiome has been implicated in increased risks of certain malignancies, including breast cancer [61]. Exercise alters the composition and derived metabolic products in the human gut microbiota by reducing the inflammatory signaling pathway induced by obesity [62,63]. Therefore, the disease-related deleterious gut microbiota profile could be modified by physical activity intervention. Overall, complex systemic changes during physical activity and structured exercise may directly inhibit breast cancer progression and improve the overall survival outcome.

## DISCUSSION

In this review, we summarize the current literature supporting various biological mechanisms whereby physical activity and exercise may influence the initiation, progression, and growth of tumors, mainly breast cancer (Figure 1; Table 1). There is strong evidence that physical activity reduces the risk of breast cancer, and the protective effect of physical activity on this type of cancer has been achieved by long-term regulation of various circulatory risk factors, such as sex hormones, metabolic hormones, and inflammatory factors. In addition, physical activity substantially alters whole-body homeostasis by inducing numerous other factors as well as by changing the gut microbiota profile. Ultimately, every cell and organ in the body might be affected by the biological perturbation induced by exercise. The potential intracellular mechanisms underlying the effects of physical activity on breast carcinogenesis include the phosphatidylinositol-3-kinase/protein kinase B and mammalian target of rapamycin signaling pathways as well as cell cycle and apoptosis [64]. However, most health-promoting benefits induced by exercise are associated with body mass index and weight loss, indicating that the level of adiposity and the percentage of fat mass are critical indicators in determining the effect of exercise.

Current physical activity recommendations for breast cancer survivors encourage following aerobic exercise routines that include 150 min per week of moderate or 75 min per week of high intensity exercise, and resistance exercise for at least 2 days per week [65-67]. However, as breast cancer is generally considered as a heterogeneous disease, the impact of physical activity may differ depending on the clinicopathologic features (e.g., tumor stage and hormone receptor status) and body composition (e.g., fat and skeletal muscle mass), eliciting diverse biological and molecular mechanisms and the varied and limited outcomes of breast cancer. In addition, breast cancer progression is influenced by the integrity and composition of the tumor microenvironment; the efficacy of exercise intervention is probably dependent on the modulation of host-tumor interaction. Moreover, even though the evidence for the benefits of physical activity in breast cancer continues to grow, most studies have not applied all the components of exercise prescription (frequency, intensity, time, and type). Recently, guidelines for academic researchers have been published for reporting exercise programs to increase clinical uptake and improve patient outcomes [68]. Therefore, further investigations should follow the Consensus on Exercise Reporting Template to understand a detailed mechanistic explanation for physical activity-dependent suppression of breast cancer growth for the transparency, consistency, and implementation of effective exercise interventions in clinical practice.

Physical activity could be considered an efficacious non-pharmacological therapy, and its promotion is probably an effective strategy in primary cancer prevention [69]. Exercise training has a significant physiological effect on IGF-1 in postmenopausal breast cancer survivors [28], and high-intensity interval training shows a remarkable effect on the expression of microRNAs in breast cancer patients undergoing hormone therapy. Although the expression of microRNAs does not change in healthy women after a 12-week exercise program, emerging evidence suggests that exercise is also directly linked to cancer progression by affecting tumor-intrinsic factors [14]. Therefore, not only exercise itself is a medicine in oncology, but it is also a critical synergistic medicine with conventional anti-cancer therapies, such as chemotherapy or hormone therapy. However, more research is needed to fully understand the direct and synergistic effects of exercise on breast cancer progression.

In conclusion, the biology of physical activity and exercise is highly complex and variable, and affects multiple organ systems via autocrine, paracrine, and endocrine factors within a crosstalk network. Thus, an understanding of these integrative mechanisms will enhance how physical activity can ultimately influence the risk and prognosis of various cancers, including breast cancer.

## Figures and Tables

**Table 1.** Summary of physical activity-dependent regulators of systemic factors involved in breast cancer risk, progression, and recurrence

Mediators affected by physical activity^*^	Ref
**Sex steroid hormones**	
Estrogen, androgen, testosterone	17-23
**Metabolic hormones**	
Insulin, leptin, IGF	24-31
**Inflammatory markers**	
CRP, TNF-α, IL-6, IL-1b, IL-7, IL-15	32-35
**Adipokines**	
Adiponectin, leptin, resistin	36-40
**Myokines**	
Myostatin, myonectin, irisin, IL-6, ANGPTL-4, MCP-1, CX3CL1, IL-8, IL-15	41-43
**Stress hormones**	
Catecholamines; epinephrine, norepinephrine, cortisol	44-46
**Others**	
Metabolites	47, 48
Reactive oxygen species	49, 50
microRNAs; miR-21, let-7a	51-53
Exosomes	54-57
Immune cells; NK cells, MDSCs	58-60
Gut microbiomes	61-63

* IGF, insulin-like growth factor; CRP, C-reactive protein; TNF, tumor necrosis factor; IL, interleukin; ANGPTL-4, angiopoietin-like 4; MCF-1, monocyte chemoattractant protein-1; CX3CL1, C-X3-C motif chemokine ligand 1; NK, natural killer cells; MDSCs, myeloid-derived suppressor cells.
